# The Poly (ADP-Ribose) Polymerase Inhibitor Veliparib and Radiation Cause Significant Cell Line Dependent Metabolic Changes in Breast Cancer Cells

**DOI:** 10.1038/srep36061

**Published:** 2016-11-04

**Authors:** Vijesh J. Bhute, Yan Ma, Xiaoping Bao, Sean P. Palecek

**Affiliations:** 1 Department of Chemical and Biological Engineering, University of Wisconsin-Madison, Madison, WI 53706, USA.

## Abstract

Breast tumors are characterized into subtypes based on their surface marker expression, which affects their prognosis and treatment. Poly (ADP-ribose) polymerase (PARP) inhibitors have shown promising results in clinical trials, both as single agents and in combination with other chemotherapeutics, in several subtypes of breast cancer patients. Here, we used NMR-based metabolomics to probe cell line-specific effects of the PARP inhibitor Veliparib and radiation on metabolism in three breast cancer cell lines. Our data reveal several cell line-independent metabolic changes upon PARP inhibition. Pathway enrichment and topology analysis identified that nitrogen metabolism, glycine, serine and threonine metabolism, aminoacyl-tRNA biosynthesis and taurine and hypotaurine metabolism were enriched after PARP inhibition in all three breast cancer cell lines. Many metabolic changes due to radiation and PARP inhibition were cell line-dependent, highlighting the need to understand how these treatments affect cancer cell response via changes in metabolism. Finally, both PARP inhibition and radiation induced a similar metabolic responses in BRCA-mutant HCC1937 cells, but not in MCF7 and MDAMB231 cells, suggesting that radiation and PARP inhibition share similar interactions with metabolic pathways in BRCA mutant cells. Our study emphasizes the importance of differences in metabolic responses to cancer treatments in different subtypes of cancers.

Breast cancer is one of the most commonly occurring cancers in women around the world^1^. Roughly 10–20% of the invasive breast cancers^1^,^2^ are triple negative breast cancers (TNBCs), i.e., they lack estrogen receptor (ER), progesterone receptor (PR) and do not overexpress human epidermal growth factor receptor 2 (HER2). This subtype of breast cancers is often associated with mutations in the BRCA1 gene which plays an important role in DNA repair via homologous recombination^3^,^4^. Due to the lack of ER, PR, and HER2, these TNBCs show poor response to hormone therapies, limiting treatment strategies. Indeed, patients with TNBCs have poorer prognosis than patients with other forms of breast cancer^1^.

Recently, poly(ADP-ribose) polymerase (PARP) inhibitors (PARPis) have shown promising anticancer activity in BRCA1 and BRCA2 mutant tumors, both as single agents and in combination with other anticancer treatments including radiation^5^,^6^,^7^. The increased susceptibility of BRCA1 and BRCA2 mutant tumors toward PARPis is thought to result from the involvement of PARP1 in DNA repair via base excision repair (BER) and homologous recombination (HR)^8^. In addition to DNA repair pathways, PARP1 also plays important roles in several cellular processes such as transcriptional regulation^9^, cell death^10^, angiogenesis^11^, and metabolism^12^,^13^. Despite the increased interest in PARPis as cancer therapeutics^5^, a detailed understanding of their effects on the aforementioned cellular processes is lacking.

Cancer metabolism plays an important role in every stage of tumor pathology^14^ and some of the earliest discoveries that identified differences between tumor and healthy cells involved differences in metabolism of glucose (e.g., the Warburg effect^15^). Recent studies have identified that multiple metabolites promote tumor growth by inhibiting apoptosis and senescence^16^ and therefore dysregulation of cellular energetics was included in the list of hallmarks of cancer^14^. Metabolomics paired with statistical analysis can be a powerful tool in biomarker discovery for cancer diagnosis, and therapeutic evaluation^17^.

In a previous study^18^, we identified several metabolic changes in MCF7 breast cancer cells in response to Veliparib (ABT-888), a potent PARPi, as well as radiation. These included significantly higher levels of NAD^+^, glutamine, myo-inositol, taurine, and sn-glycero-3-phosphocholine (GPC), and significantly lower levels of lactate, alanine, pyruvate, phosphocreatine after one day of PARPi treatment. Radiation alone led to significant depletion of several amino acids and increases in taurine and phosphocholine two days after the radiation treatment. In this study, we sought to identify the cell line-independent effects of PARP inhibition (PI) on cancer cell metabolism and compare these effects with the metabolic responses elicited by radiation. We used three breast cancer cell lines, HCC1937, MDAMB231 and MCF7, with differences and similarities between genotypes and phenotypes of these lines summarized in Table 1. Using NMR metabolomics, we show that different breast cancer lines share some metabolic responses to PI. Pathway topology and enrichment analysis on the metabolic responses after PI revealed significant enrichment in several common pathways including protein synthesis, nitrogen metabolism, and taurine metabolism. However, the majority of the metabolic responses to PI were cell line dependent. When we compared the metabolic responses to radiation, our data indicate that only the BRCA mutant cell line, HCC1937, showed extensive metabolic responses 24 hours after the radiation treatment as compared to an untreated control, and shared some similarity in metabolic changes with those elicited by PI. Together, our data suggest significant cell line-dependent effects on metabolism due to PARP inhibition and radiation in breast cancer cells.

## Results and Discussion

### DNA damage activates PARP to a greater extent in HCC1937 cells than in MDMAB231 cells and MCF7 cells

**Figure 1:**
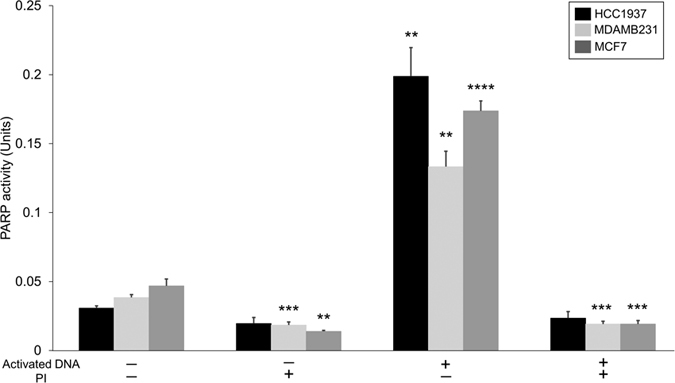
Effect of PARP inhibition on basal activity (-activated DNA) and on activation (+activated DNA) in breast cancer cells. PARP activity increased more than 6-fold in HCC1937 cells and 3.5-fold in MCF7 and MDAMB231 cells in the presence of activated DNA relative to respective basal activities. PARP was inhibited using 50 μM ABT-888 which led to about 80% reduction in PARP activity compared to the DMSO control in the three cell lines. Statistical analysis is performed on samples from three biological replicates using two-tailed t-test for comparing the PARP activity in each cell line relative to their basal levels (−/−). The error bars represent standard deviations. *p < 0.05, **p < 0.01, ***p < 0.001, ****p < 0.0001 relative to respective basal levels (−/−).

Triple negative breast cancer cells exhibit poor response to hormonal therapy, thus their treatment typically involves chemotherapy, radiation, and/or surgery. The HCC1937 cell line is homologous for the mutant BRCA gene, while the MDAMB231 and MCF7 cell lines have wild type BRCA gene expression. The protein encoded by the BRCA gene plays an important role in homologous recombination-mediated DNA repair. Recent studies have shown that BRCA mutant^5^,^6^,^7^ and HER2 overexpressing breast cancer cells^19^ show increased sensitivity towards PI both as a single agent and in combination with radiation. This can be partially explained by the overactivation of PARP in cells having inefficient HR machinery^20^. This elevated sensitivity to PI by HER2 overexpressing cells was also observed in our *in vitro* assays to measure activity of PARP in the presence or absence of exogenous broken DNA strands (activated DNA). The HCC1937 cell line exhibited a 6.5 fold increase in PARP activity in presence of activated DNA vs. a 3.5 fold increase in MDAMB231 and MCF7 cell lines (Fig. 1). PI led to over 85% inhibition in PARP activity (in the presence of activated DNA) in the three cell lines.

### Breast cancer cell lines show BRCA-dependent metabolic responses to radiation

We investigated the metabolic responses induced by radiation and PI in cell lines expressing mutant and wild-type BRCA and different HER2 levels. We chose a radiation dosage (8 Gy) which resulted in ~70–80% survival in these cell lines 24 hours after radiation treatment (Supplementary Fig. 1a). We also tested different concentrations of the potent PI Veliparib (ABT-888) on the three cells lines and chose 50 μM, which led to ~80% inhibition of PARP activity in presence of damaged DNA, assayed using a chemiluminiscent PARP activity assay (Trevigen Inc.) (Supplementary Fig. 1b).

**Figure 2:**
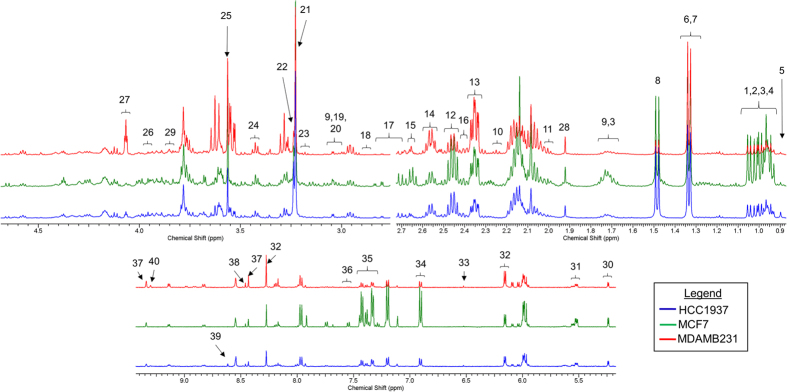
Representative NMR spectra for annotated peaks of intracellular metabolites. 1: Isoleucine, 2: Valine, 3: Leucine, 4: 2-oxoisocaproate, 5: Pantothenate, 6: Lactate, 7: Threonine, 8: Alanine, 9: Lysine, 10: 2-aminoadipate, 11: Proline, 12: Glutamine, 13: Glutamate, 14: Glutathione, 15: Methionine, 16: Pyroglutamate, 17: Aspartate, 18: Asparagine, 19: Creatine, 20: Creatine phosphate, 21: O-phosphocholine, 22: Sn-glycero-3-phosphocholine, 23: Beta-alanine, 24: Taurine, 25: Glycine, 26: Serine, 27: Myo-inositol, 28:Acetate, 29: Sorbitol, 30: Glucose, 31: UDP-GlycNac, 32: ATP, 33: Fumarate, 34: Tyrosine, 35: Phenylalanine, 36: Tryptophan, 37: NAD+, 38: Formate, 39: AMP, 40: 1-methylnicotinamide.

**Figure 3:**
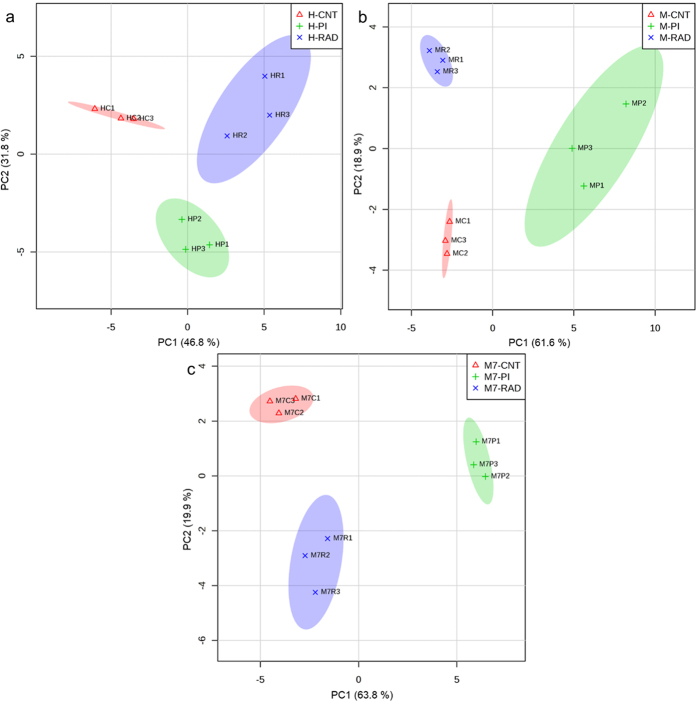
Principal component analysis of metabolite concentrations in (**a**) HCC1937, (**b**) MDAMB231 and (**c**) MCF7 cell lines. The cells were cultured in triplicate to ~80% confluence and treated with 50 μM ABT-888 (or DMSO) in fresh medium. For radiation treatment, cells were preconditioned with fresh medium for 24 hours prior to radiation treatment with 8 Gy dosage. The samples were collected as described in the methods and analyzed using ChenomX Profiler. Metabolite concentrations were normalized and auto-scaled prior to PCA using MetaboAnalyst. Each data point represents a biological sample and ellipses indicate the 95% confidence intervals. Abbreviations: PI: PARP inhibition, RAD: radiation, H: HCC1937, M: MDAMB231, M7: MCF7, CNT: control.

Representative annotated NMR spectra for the three cell lines are shown in Fig. 2 and the metabolites identified in any of the cell lines are shown in Supplementary Table 1. The metabolites are classified into different groups based on either their functions or chemical compositions. Metabolites with low abundance in all the three cell lines are included in the “others” group. We performed principal component analysis (PCA) on the complete dataset by including the three breast cancer cells (Supplementary Fig. 2) to study global metabolic profiles. Each data point on the PCA scores plot indicates an individual biological sample, and the x-axis represents the variance captured by the first principal component (PC) and the y-axis represents the variance captured by the second PC and z-axis represents the variance captured by the third PC. We observe that the differences between the metabolic profiles of breast cancer cells are highly dominant and significantly greater in comparison to the effects of drug themselves (Supplementary Fig. 2). In order to investigate the effects of drugs, we focus on each cell line independently (Fig. 3 and Supplementary Fig. 3). We performed principal component analysis (Fig. 3) and hierarchical clustering (Supplementary Fig. 3) on the concentrations of different metabolites in the three breast cancer cell lines in response to radiation and PI.

We observed that radiation-treated HCC1937 cells clustered separately from control HCC1937 cells (Fig. 3a). Also, the separation along the 1^st^ PC explained the majority of the variance (>46%) in the data indicating that radiation induced significant changes in metabolism in HCC1937 cells. In contrast, radiation-treated MDAMB231 and MCF7 cells separated from non-treated controls along the 2^nd^ PC, which explained 18–20% of the variance in the data, indicating radiation induced relatively minor differences in metabolite fractions in these cell lines (Fig. 3b,c). PI on the other hand, led to significant changes in the metabolic response in all three cell lines.

### Radiation reduced glutathione levels in HCC1937 cells and altered amino acid metabolism in the three breast cancer cell lines

**Figure 4:**
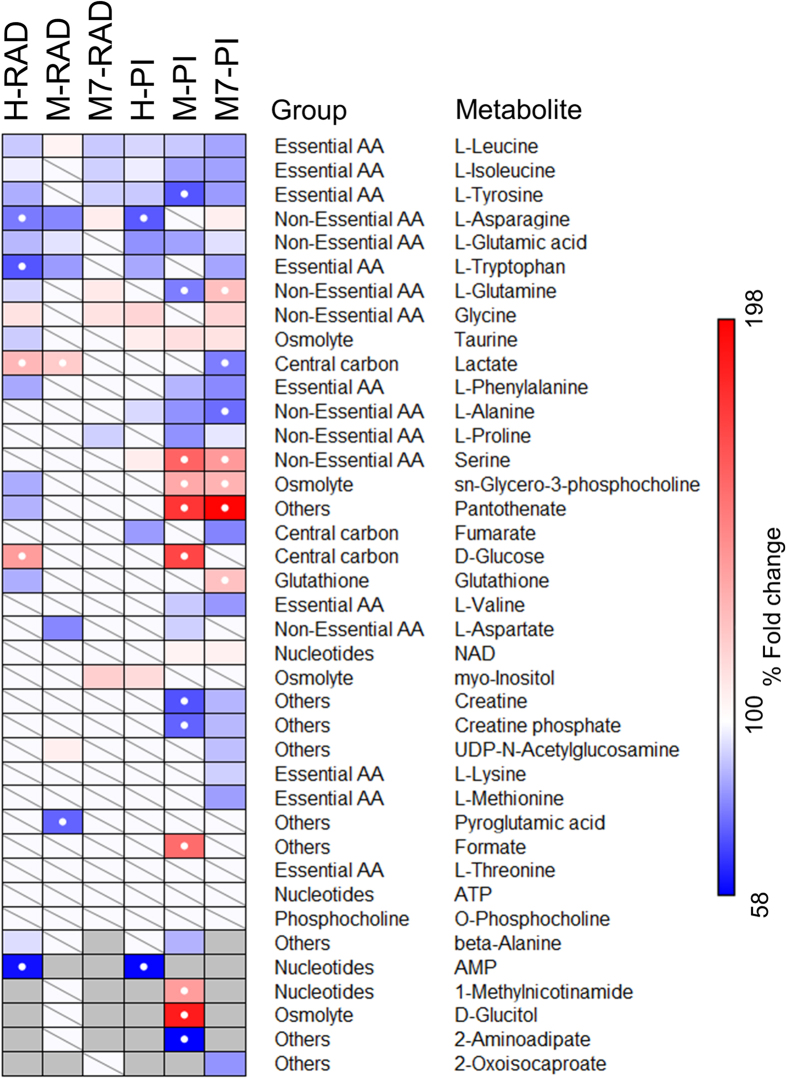
Metabolic fold changes (%) due to radiation or PI relative to control in HCC1937, MDAMB231 and MCF7 cells. Statistical analysis was performed using ANOVA with Tukey’s HSD as the post-hoc method to identify significantly affected metabolites (FDR < 0.05) due to radiation or PI relative to control in the breast cancer cells. The non-significant changes are indicated with white boxes with a diagonal line while the metabolites which were not detected in a cell line are shown in grey. Metabolites which exhibited a fold change of more than 20% are shown with a white dot. Abbreviations: PI: PARP inhibition, RAD: radiation, H: HCC1937, M: MDAMB231, M7: MCF7.

**Figure 5:**
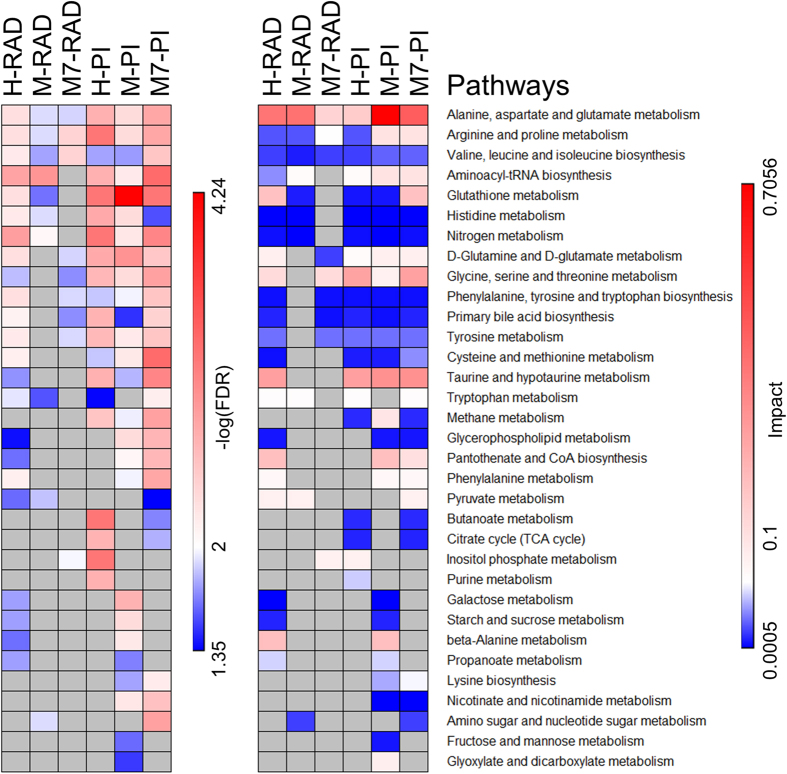
Pathway analysis to identify significantly enriched metabolic pathways due to radiation or PARP inhibition relative to control in HCC1937, MDAMB231 and MCF7 cells. The metabolites identified to be significantly affected (FDR < 0.05) from ANOVA were selected as inputs for pathway enrichment and topology analysis. Global test was used for identifying enriched pathways and relative betweenness centrality was used to evaluate the importance of the metabolites in the pathway. The pathways were considered to be significantly enriched relative to respective controls in the three cell lines if a) FDR < 0.05 and b) the number of metabolite hits in the pathway >1. The heatmap on the left indicates the –log(FDR) for the significantly enriched pathways (higher enrichment: red) and the heatmap on the right shows the corresponding impact score for each pathway. Grey boxes indicate no significance enrichment in the pathways (FDR > 0.05 and/or number of hits <2). Abbreviations: PI: PARP inhibition, RAD: radiation, H: HCC1937, M: MDAMB231, M7: MCF7.

We used ANOVA with Tukey’s HSD as the post-hoc method to identify significantly affected metabolites (FDR ≤ 0.05) upon radiation and PI (Fig. 4). As was observed in our previous study^18^, radiation led to depletion of several amino acids including isoleucine, leucine, tyrosine and proline and increases in glutamine, glycine, asparagine and myoinositol relative to untreated control MCF7 cells. Arginine and proline metabolism showed significant enrichment and impact (FDR = 0.004, Impact = 0.1) in MCF7 cells treated with radiation (Fig. 5). Pathway analysis also indicates that inositol phosphate metabolism was significantly enriched exclusively in MCF7 cells after the radiation treatment.

In MDAMB231 cells however, we observed distinct and fewer metabolic changes due to radiation relative to control (Fig. 4). We observed an accumulation of lactate and depletion of tryptophan, glutamate, asparagine, aspartate and pyroglutamate. Depletion in amino acids can affect protein biosynthesis pathways and aminoacyl tRNA biosynthesis showed significant enrichment in MDAMB231 cells relative to control (FDR = 0.0012, Impact = 0.113). Amino sugar metabolism was distinctly enriched after radiation treatment relative to control (FDR = 0.012, Impact = 0.026) in MDAMB231 cells (Fig. 5).

The HCC1937 cell line showed depletion in amino acids, except for glycine which accumulated, after radiation treatment relative to control (Fig. 4). Both glucose and lactate showed more than 20% fold change and were significantly higher relative to untreated control in HCC1937 cells. A study showed an increased glucose uptake after irradiation in HCC1937 tumor spheroids^21^. An increase in glucose transport after the induction of stress^22^,^23^ can explain the changes in intracellular glucose levels in HCC1937 cells after radiation treatment.

Glutathione also showed significant reduction after radiation treatment in HCC1937 cells suggesting an increased scavenging and formation of glutathione disulfides due to radiation induced oxidative stress^24^. Glutathione metabolism was found to be significantly enriched with a high impact score (FDR = 0.005, Impact = 0.25), after radiation treatment in HCC1937 cells (Fig. 5). Pantothenate (vitamin B_5_), a precursor to coenzyme A (CoA) synthesis, also showed significant reduction after radiation relative to untreated control cells. Reduction in pantothenate could be due to a reduced uptake and/or increased CoA synthesis. CoA also plays an important role during oxidative stress and can participate in reactions to replenish reduced glutathione^24^. Pantothenate and CoA biosynthesis pathway were also enriched exclusively in HCC1937 cells (FDR = 0.023, Impact = 0.253) after radiation treatment.

Significant reduction in both essential and non-essential amino acids (except for glycine) indicates either an increase in protein synthesis or reduced uptake of these metabolites after radiation treatment. A study by Braunstein *et al*.^25^ supports the increased protein synthesis hypothesis, although they observed this in untransformed MCF10A cells. A recent study by Hosios *et al*.^26^ showed that the majority of the cell mass in proliferating cells was derived from non-glutamine amino acids. Changes in amino acid pools can therefore affect the abundance of carbon and nitrogen for these cells. Aminoacyl t-RNA biosynthesis was found to be significantly enriched (FDR = 0.0015, Impact = 0.056) in HCC1937 cells after radiation treatment. Other metabolites which also showed significant reduction after radiation treatment were GPC and beta-alanine, both of which are known to have osmoregulatory functions^27^,^28^. Glycerophospholipid metabolism, phenylalanine metabolism, beta-alanine metabolism and propanoate metabolism were significantly enriched and distinct from those which were modulated in either MDAMB231 or MCF7 cells after radiation treatment. Galactose metabolism and starch and sucrose metabolism were also significantly affected due to radiation in HCC1937 cells but had a very low impact scores (Fig. 5).

Alanine, aspartate and glutamate metabolism was significantly enriched after radiation treatment relative to control in the three cell lines and also had a very high impact score (Fig. 5). Other pathways which were also significantly enriched after radiation common to the three cell lines include arginine and proline metabolism and valine, leucine and isoleucine metabolism. Aminoacyl tRNA biosynthesis, glutathione metabolism were significantly enriched due to radiation treatment in both HCC1937 and MDAMB231 cells, but the impact and –log(FDR) were higher for HCC1937 cells, suggesting an increased enrichment and impact in these cells relative to MDAMB231 cells. Overall our data suggest increased oxidative stress, indicated by reduction in glutathione, in the BRCA mutant HCC1937 cells due to radiation treatment relative to untreated control and alteration in amino acid metabolism in all the three cells lines 24 hours after radiation treatment.

### PI-induced increase in NAD^+^ concentrations correlates with a reduction in creatine in MCF7 and MDAMB231 cells

MCF7 and MDAMB231 cells showed an increase in NAD^+^ concentration after PI. NAD^+^ concentrations are highly regulated^29^ and it serves as the substrate for PARP during poly-ADP ribosylation^30^. We expected to observe higher NAD^+^ levels in all three cell lines due to inhibition of PARP activity, but, NAD^+^ levels were not significantly affected in HCC1937 cell line upon PI relative to control (Supplementary Fig. 4). A possible explanation is that even after PI, the residual PARP activity in HCC1937 cells led to similar NAD^+^ consumption as control treated cells. This is evident from Fig. 1 where in the absence of exogenous damaged DNA (no activated DNA), the PARP activity in HCC1937 cells was not significantly inhibited in the presence of PARPi, while, it was significantly inhibited by 50% (p < 0.001) and 73% (p < 0.01) in MDAMB231 and MCF7 cells respectively. So, the net change in basal PARP activity was more pronounced in MDAMB231 and MCF7 cells. Since PARPi competes with NAD^+^ for binding to PARP thereby blocking PARP’s ADP-ribosylation activity, increased net change in PARP activity in both MCF7 and MDAMB231 cells leads to increase in NAD^+^ concentration. The net change in basal PARP activity due to PARPi in HCC1937 cells was not significant which correlated with no significant change in NAD^+^ concentration. A possible explanation for this differential effect of PARPi could be due to differences in the basal PARP activity itself in the three cell lines. HCC1937 cells had significantly lower basal PARP activity (Fig. 1). Following treatment with PARPi, the PARP activity in HCC1937 cells was similar to that in MCF7 and MDAMB231 cells.

The reduced basal PARP activity in HCC1937 cells could be attributed to either reduced recruitment of PARP to DNA damage sites or reduced endogenous DNA damage in HCC1937 cells as compared to MCF7 and MDAMB231 cells. The latter possibility is more likely as the replicative stress is reduced in HCC1937 cells due to slower growth rate as compared to MCF7 and MDAMB231 cells (Table 1).

NAD^+^ plays an important role in mitochondrial metabolic homeostasis^29^ and a reduced impact of PARPi on NAD^+^ (in absence of exogenous DNA damage) in HCC1937 cells may explain a relatively reduced effect on metabolism due to PI in HCC1937 cells as compared to that observed in MDAMB231 and MCF7 cells. We therefore explored the unique metabolic changes which were common to MDAMB231 and MCF7 cells to gain insights into the metabolic changes that correlated with NAD^+^ accumulation.

**Figure 6:**
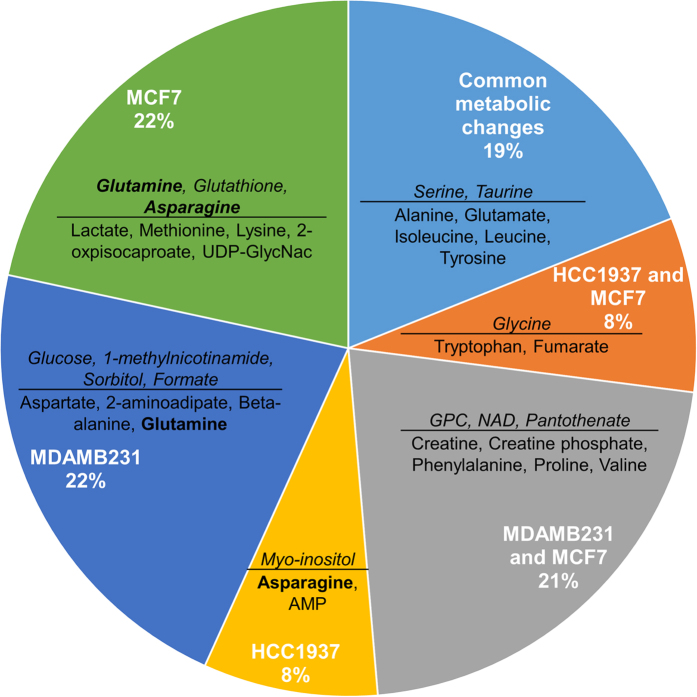
Pie chart representing the similarities and differences in metabolic responses due to PI in the HCC1937, MDAMB231 and MCF7 cells. The metabolites which exhibited significant increases (FDR < 0.05, ANOVA) due to PI relative to control for each cell line are shown in italics (numerator) and the metabolites which showed significant decreases (FDR < 0.05, ANOVA) due to PI relative to control for each cell line are in the denominator. Bold indicate the metabolites which increased in one cell line but decreased in another (glutamine and asparagine). Abbreviations: GPC: sn-glycero-3-phosphocholine.

Creatine and phosphocreatine were depleted after PI in MDAMB231 and MCF7 cells relative to respective controls (Figs 4 and 6). Creatine and phosphocreatine can shuttle ATP and provide energy to compensate for reduced ATP levels^31^. In a previous study^18^, we observed that NAD^+^ depletion due to an alkylating agent, methyl methansulfonate (MMS), led to significantly increased concentrations of creatine in MCF7 cells. Additionally, the restoration of NAD^+^ concentrations by a combination of MMS and PI led to a significant reduction in creatine concentration^18^. This suggests that there exists a negative correlation of creatine with NAD^+^ concentration. We also showed that PI alone led to significant reduction in phenylalanine and valine and an increase in GPC in MCF7 cells^18^. These changes were also observed here in both MDAMB231 and MCF7 cells, further supporting that these metabolite changes correlate with accumulation of NAD^+^.

### PI led to accumulation of taurine and serine in a cell line-independent manner

Even though the NAD^+^ concentration was not elevated in HCC1937 cells, there were several metabolites which showed similar metabolic changes following PI in the three breast cancer cell lines (Figs 4 and 6). PI led to accumulation of taurine and serine, and reduction in amino acids including alanine, glutamate, isoleucine, leucine, and tyrosine in the three breast cancer cell lines. Correspondingly, taurine and hypotaurine metabolism; glycine, serine and threonine metabolism; aminoacyl tRNA biosynthesis; and alanine, aspartate and glutamate metabolism showed significant enrichment and high impact scores when comparing PI relative to untreated controls in the three cell lines.

These cell line-independent changes to PI correspond to conserved effects and can imply novel DNA independent functions of PARP in the cells. For example, taurine is an osmoregulatory metabolite which also plays an antioxidant role by suppressing nitrosative stress^32^ and regulates the mitochondrial protein synthesis and superoxide generation^33^. Consistent increases in taurine concentrations after PI suggest an improved ability to reduce the oxidative and nitrosative stress. PARP activation has previously been associated with increase in nitrosative stress^10^ and this could be potentially mediated by altering taurine concentrations. Additionally, reduction in several amino acids could indicate increased protein synthesis which has been shown to be regulated by taurine^33^.

An increase in serine concentration after PI could suggest an increased flux through serine biosynthesis and/or uptake pathways or a reduced flux through the serine degradation pathway. Serine can serve as substrate for one carbon metabolism and support purine synthesis^34^. Therefore, PI could potentially alter the purine biosynthesis pathway or affect serine biosynthesis and/or uptake in breast cancer cells.

### PI induced significant cell line dependent metabolic changes in breast cancer cells

MCF7 and MDAMB231 cells shared several similarities (40%) in metabolic changes due to PI relative to respective controls (Fig. 6). HCC1937 (BRCA mutant) cells on the other hand exhibited fewer metabolic changes due to PI (Fig. 4). We observed that only 19% of the metabolic changes were shared between all the three cell lines and the majority (52%) of the total significant changes were cell line dependent (Fig. 6).

There were significantly higher levels of different osmolytes after PI relative to control in the three cell lines. While taurine was elevated in all three cell lines, sorbitol was increased only in MDAMB231 cells and myo-inositol accumulated only in HCC1937 cells. GPC concentration increased in both MCF7 and MDAMB231 cells. In addition to their roles in osmoregulation, these metabolites also affect signaling as well as metabolic pathways. Associated metabolic pathways which showed significant enrichment in specific cell lines include inositol phosphate metabolism in HCC1937 cells; starch and sucrose metabolism; and fructose and mannose metabolism in MDAMB231 cells; and glycerophospholipid metabolism in both MDAMB231 and MCF7 cells (Fig. 5).

These cell lines also showed depletion of several amino acids due to PI relative to control. Aspartate and glutamine were depleted in MDAMB231 cells while asparagine was depleted in HCC1937 cells, and methionine and lysine showed reduction in concentrations in MCF7 cells. In addition to their roles in protein biosynthesis^26^, these amino acids can also support energy requirements by providing carbon for the citric acid cycle^35^,^36^ and participate in multiple anabolic pathways including purine synthesis and glutathione metabolism. The aminoacyl tRNA biosynthesis pathway showed significant enrichment in all three cell lines due to PI despite the changes in the actual amino acids which were depleted in these cells.

### PI induced metabolic changes in MDAMB231 cells support reduced oxidative stress relative to control

MDAMB231 cells showed an increase in 1-methylnicotinamide (1-MN) and a reduction in 2-aminoadipate (aminoadipic acid) and beta-alanine due to PI relative to control. 1-MN and aminoadipic acid metabolites were below the detection limit of NMR in MCF7 and HCC1937 cells. The difference in the abundance of these metabolites suggests a difference in the specific metabolic pathways. 1-MN belongs to nicotinate and nicotinamide metabolic pathways and is a product of nicotinamide N-methyltransferase (NNMT) activity on S-adenosylmethionine (SAM) and niacinamide. SAM plays a critical role as a methyl donor for methylation of DNA, RNA, phospholipids, proteins, creatine and other molecules^37^. Increased activity of NNMT is also associated with various aggressive human cancers and its contribution to tumorigenesis is linked with its ability to negatively affect the methylation potential by reducing SAM, thereby altering the epigenetic state^38^. A recent study found that overexpression of NNMT can promote growth and enhance migration and invasion capacities in pancreatic cancer cells^39^. NNMT activity can also help in metabolizing drugs and other xenobiotic compounds in the liver^40^. PI led to an increase in 1-MN concentration, suggesting that NNMT activity may be affected by PI. Increased NNMT activity in MDAMB231 cells could indicate an alternate mechanism for metabolizing chemotherapeutic drugs and resistance or differential response.

We found that PI led to more than 40% reduction (fold change: 0.57, FDR = 0.004) in aminoadipic acid concentration in MDAMB231 cells (Fig. 4). Aminoadipic acid is an intermediate product of the lysine degradation pathway and a recent study^41^ found that its concentration was elevated in prostate biopsies from prostate cancer patients. Importantly, it was also found to be associated with tumor recurrence and could be used in a model with tumor stage and Gleason score for prediction of biochemical recurrence in prostate cancer patients^41^. Previous studies have shown that aminoadipic acid can serve as a biomarker for oxidative stress^42^,^43^. A study by Sell *et al*.^44^ showed that in aging human skin, aminoadipic acid was found to be marker of protein carbonyl oxidation. A reduction in aminoadipic acid due to PI could be due to reduced PARP activation-mediated oxidative or nitrosative stress^10^.

Beta-alanine is a product of proteolytic degradation of di-peptides including carnosine, anserine, balenine, and pantothenate (vitamin B_5_). Pantothenate was increased in MDAMB231 cells due to PI (Figs 4 and 6). Reduction of beta-alanine and an increase in pantothenate concentration in MDAMB231 cells due to PI could be associated with reduced degradation of pantothenate. Pathway topology analysis revealed beta-alanine metabolism and pantothenate and CoA biosynthesis pathways to be significantly enriched due to PI in MDAMB231 cells (Fig. 5).

### PI in MCF7 cells can lead to changes in mitochondrial metabolic pathways

Glutathione concentration was significantly increased due to PI in MCF7 cells. Glutathione plays a critical role in scavenging reactive oxygen species (ROS), thereby reducing the oxidative stress^45^. An increase in glutathione concentration in MCF7 cells indicates reduced oxidative stress due to PI. Lactate concentration was significantly reduced due to PI relative to control in MCF7 cells. An increase in NAD^+^ concentration due to PI can affect mitochondrial metabolism and the glycolytic flux^29^. This may potentially affect the lactate concentration in MCF7 cells. Several other amino acids showed significant differences, including increases in glutamine and asparagine, and decreases in methionine and lysine. Glutamine and asparagine can provide carbon for the citric acid cycle^35^,^36^ and an increase in their concentration due to PI could be attributed to NAD^+^ induced mitochondrial energy homeostasis. UDP-N-acetylglucosamine (UDP-GlycNAc) concentration was reduced due to PI in MCF7 cells. UDP-GlycNac is a nucleotide sugar which plays varied functions, including pyrimidine biosynthesis and glycosylation of proteins. It is the final product of the hexosamine biosynthesis pathway which activates O-GlcNAcylation, thereby affecting histone remodeling, transcription, proliferation, apoptosis, and proteasomal degradation^46^. Pyruvate metabolism, citric acid cycle and amino sugar and nucleotide metabolism were significantly enriched due to PI in MCF7 cells (Fig. 5).

### Radiation and PI induce similar metabolic changes relative to control in HCC1937 cells

Finally, we compared the metabolic changes induced by radiation versus those by PI relative to control in the three cell lines (Figs 3, 4 and 5). It is evident from the PCA that the radiation and PI showed distinct metabolic changes in MCF7 and MDAMB231 cells indicated by the separation along the 1^st^ PC (Fig. 3b,c). But in HCC1937 cells both radiation and PI separated along the 2^nd^ PC which explains 32% of the variance (Fig. 3a). These data imply that the majority of the metabolic changes observed in radiation and PI relative to control were similar in HCC1937 cells. This is further supported by the statistical analysis using ANOVA which showed that both radiation and PI induced reduction in several amino acids including alanine, asparagine, glutamate, leucine, tryptophan, tyrosine and increased glycine concentrations relative to untreated control in HCC1937 cells (Fig. 4). Pathway enrichment and topology analysis showed the similarity in the impact score of the majority of the pathways which were significantly enriched after radiation or PI in HCC1937 cells (Fig. 5). This suggests a similarity in the effect on metabolism, most likely due to activation of similar pathways^18^, in HCC1937 cells.

## Conclusions

Cancer cells accumulate extensive mutations, among other changes, to survive and proliferate in an unregulated manner. The signaling and metabolic activity of different types of cancer is therefore distinct and so is the response to different anticancer therapies. In this study, we used a metabolomics approach to study the similarities and differences in metabolic responses of three subtypes of breast adenocarcinoma cell lines to radiation and PI. Radiation induced significant changes in HCC1937 cells which were similar to PI relative to control. These included reduction in amino acids, suggesting an alteration in protein synthesis due to radiation or PI. PI led to increased NAD^+^ concentration, which also correlated with a decrease in creatine concentration, in MCF7 and MDAMB231 cells. The effect of PI on metabolism was pronounced in MCF7 and MDAMB231 cells relative to HCC1937 cells and is most likely due to changes in NAD^+^ concentration due to PI. PI led to cell line independent changes including accumulation of taurine and serine. Further analysis revealed several other osmolytes and antioxidants to be significantly increased in either of the three cell lines. These metabolic changes can be linked to suppression of PARP-induced nitrosative stress^10^. PI also led to significant decreases in lactate and UDP-GlycNAc, and increases in glutamine and asparagine in MCF7 cells. These metabolic changes support changes in mitochondrial energy pathways due to PI in MCF7 cells. Our data suggest that PI can induce significant cell line dependent metabolic changes and, except for HCC1937 cells, these changes are distinct from radiation-induced metabolic changes. We also identified major pathways which were affected due to radiation or PI in these breast cancer cells. This study provides novel insights into both cell line specific and cell line independent effects of PI in breast cancer cells which can be useful in understanding differences in response in different types of breast cancer patients.

## Methods

### Cell Culture

HCC1937, MDAMB231 and MCF-7 cells were obtained from ATCC and cultured in Dulbecco’s Modified Eagle Medium (DMEM, high glucose, Thermo Fisher Scientific) supplemented with 10% fetal bovine serum (FBS, Thermo Fisher Scientific) and 1% penicillin/streptomycin (P/S, Thermo Fisher Scientific) in 5% CO_2_ and 37 °C in T-75 flasks until they reached sub-confluence (~80% or ~8 million cells). The medium was changed to fresh medium with ABT-888 (or DMSO) and the cells were treated with radiation (8 Gy at 216 rad/min) after 24 hours. The cells were quenched 24 hours after the radiation treatment.

### Radiation Treatment

To identify appropriate dosage of radiation treatment, HCC1937, MDAMB231 and MCF-7 cells were seeded in 6 well plates and after reaching ~80% confluence, cells were irradiated with a ^137^Cs source (J. L. Sheppard Mark I unit) at a dose rate of 216 rad/min with a total dose of 4, 8 or 12 Gy and the cells were counted after 24 hours using a hemocytometer (Supplemental Fig. 1a). 8 Gy treatment for 24 h led to a ~80% survival and the samples were collected for NMR analysis.

### Sample Preparation for NMR

NMR sample collection was performed as described in Bhute and Palecek^18^. Briefly, the cells were washed twice with ice-cold PBS (pH 7.4) and then the cells were quenched using 3 ml methanol as it gives better extraction efficiency and provides rapid quenching as compared to other methods^47^. The cells were then detached using a cell scraper and pipetted into a 50 ml centrifuge tube. A dual phase extraction procedure adopted from Martineau *et al*.^48^ was used to extract the intracellular metabolites. Briefly, the quenched cells were suspended in a mixture of chloroform, methanol and water in the ratio of 6:6:5.4 to make a final volume of 17.4 ml. After centrifugation for 15 min at 3600 rpm, only the upper aqueous layer was used for further analysis and the organic phase was discarded. The samples were dried at 30 °C using a centrifugal vacuum concentrator and stored at −80 °C until NMR analysis.

Prior to NMR analysis, the samples were reconstituted in 0.6 ml of 0.1 M phosphate buffered D_2_O (pH = 7.0) solution containing 0.5 mM 3-trimethylsilyl-propionate-2, 2, 3, 3,-d4 (TMSP, δ = 0.0 ppm) as an internal standard and 0.2% w/v sodium azide. The samples were centrifuged at 18,000 g for 10 min and 550 μL of the supernatant was transferred to 5 mm NMR tubes (Norell Inc.).

### NMR Acquisition

^1^H NMR spectra were recorded at 298 K on a Bruker Avance III equipped with a 5 mm cryogenic probe operating at 500 MHz (11.74 T). 1D spectra were acquired using standard NOESYPR1D pulse sequence (RD-90°-t1-90°-tm-90°-acquire) with a relaxation delay of 1 s, a mixing time of 100 ms and a pre-scan delay of 30 μs. Each spectrum consisted of 128 transients or free induction decays (FIDs) collected into 48 K complex data points with a spectral width of 12 ppm and an acquisition time of 4 s. Prior to Fourier transformation, the FIDs were zero-filled to 128 k data points and multiplied by an exponential window function (LB = 0.5 Hz). The chemical shifts were referenced to the TMSP peak (δ = 0 ppm), using TopSpin^TM^ software (version 3.1, Bruker).

### Data Processing and Statistical Analysis

Spectra were exported to an ACD/1D NMR Processor (Advanced Chemistry Development) for phasing, baseline correction, and solvent region removal (water: 4.7–5.1 ppm and DMSO: 2.72–2.75 ppm). The peaks were annotated through the HMDB^49^ and Metabohunter^50^ and targeted profiling^51^ was accomplished using ChenomX NMR Suite Profiler (version 7.7, ChenomX Inc.). The concentrations were referenced to a TMSP concentration of 0.5 mM. Over 95% of the peaks were assigned to metabolites available in the ChenomX library and the quantified metabolite concentrations were exported to an Excel file. First, the metabolites with low confidence in fitting with confounding peaks due to high overlap or very low abundance were excluded from the analysis. These included citrate, guanidoacetate, isobutyrate, and malate. Next, we excluded the metabolites which showed a coefficient of variance greater than 0.3 in all the samples for a cell line. Acetate was removed based on this criterion. The concentration data matrix was further normalized by the total concentration of metabolites in each sample to evaluate the metabolite fractions and also to account for the differences in efficiencies of extraction.

The concentration data table was processed for statistical analysis using MetaboAnalyst^52^,^53^ and GENE-E^54^. Hierarchical clustering was performed on auto-scaled concentration data (mean centering followed by dividing by the standard deviation) and one way analysis of variance (ANOVA) with Tukey’s HSD as post hoc method was used to identify significantly altered metabolites (FDR < 0.05) in each cell line (Supplementary file 1). Pathway topology analysis was performed in MetaboAnalyst’s pathway analysis module on the significantly altered metabolite for each cell line using global test algorithm for pathway enrichment (adjusted for multiple testings) and relative betweenness centrality to assess metabolite importance. The *Homo sapiens* library was used for analysis and the metabolic pathways with an impact score greater than 0, at least two metabolite hits and FDR less than 0.05 were considered to be significantly enriched. The raw and normalized concentration data from the pathway topology analysis are available in Supplementary File 1^55^. The NMR spectral files and processed JCAMP files are available on the MetaboLights database, http://www.ebi.ac.uk/metabolights/MTBLS337.

### PARP Activity Assay

Activity of poly (ADP-ribose) polymerase was measured using the Universal Chemiluminescent PARP assay Kit (Trevigen Inc.) according to manufacturer’s instructions. Cells were grown in 6 well plates with medium alone or medium containing different concentrations of ABT-888 for 24 h. Cells were then washed twice with PBS and trypsinized to detach from the surface. Medium was added to the cells after detachment and centrifuged to form cell pellets. Medium was aspirated and the cells were rinsed with PBS and centrifuged again. The cell pellet was lysed on ice for 15 min with occasional vortexing in ice cold MPER reagent containing Complete Protease inhibitor (Roche) and 150 mM NaCl. The lysates were centrifuged at 18,000 g at 4 °C for 10 min and stored at −80 °C until further analysis. Total protein content was calculated using Bradford protein dye reagent (Bio-Rad, Pierce) and 20 μg of the protein was used for the assay. The activated DNA was excluded in some samples to study the basal activity of PARP in the cell lines.

## Additional Information

**How to cite this article**: Bhute, V. J. *et al*. The Poly (ADP-Ribose) Polymerase Inhibitor Veliparib and Radiation Cause Significant Cell Line Dependent Metabolic Changes in Breast Cancer Cells. *Sci. Rep.* **6**, 36061; doi: 10.1038/srep36061 (2016).

**Publisher’s note:** Springer Nature remains neutral with regard to jurisdictional claims in published maps and institutional affiliations.

## Supplementary Material

Supplementary Information

## Figures and Tables

**Table 1:** Properties of the breast cancer cell lines used in the current study.

Property	HCC1937	MDAMB231	MCF7
Population doubling time	~52 hr	~32 hr	~30 hr
BRCA	Mutant	WT	WT
ER/PR/(Her2/neu overexpression)	−/−/−	−/−/−	+/+/−
Morphology or type (Vimentin expression)^56^	Epithelial (−)	Mesenchymal (+)	Epithelial (−)
PI3K signaling mutations^57^	PTEN deletion	Braf	PIK3CA activating mutation
