# Supplementary material for: The Poly (ADP-Ribose) Polymerase Inhibitor Veliparib and Radiation Cause Significant Cell Line Dependent Metabolic Changes in Breast Cancer Cells
Source: Sci Rep. 2016 Nov 4;6:36061. doi: 10.1038/srep36061 (PMC5095763; doi:10.1038/srep36061)
Supplement: Supplementary Information [file srep36061-s1.pdf]

Supplementary Information

The Poly (ADP-Ribose) Polymerase Inhibitor Veliparib and Radiation Cause Significant Cell Line Dependent Metabolic Changes in Breast Cancer Cells

Vijesh J. Bhute, Yan Ma, Xiaoping Bao, and Sean P. Palecek*

Department of Chemical and Biological Engineering, University of Wisconsin-Madison, Madison, WI-53706

* Corresponding author

Email: sppalecek@wisc.edu (SPP)

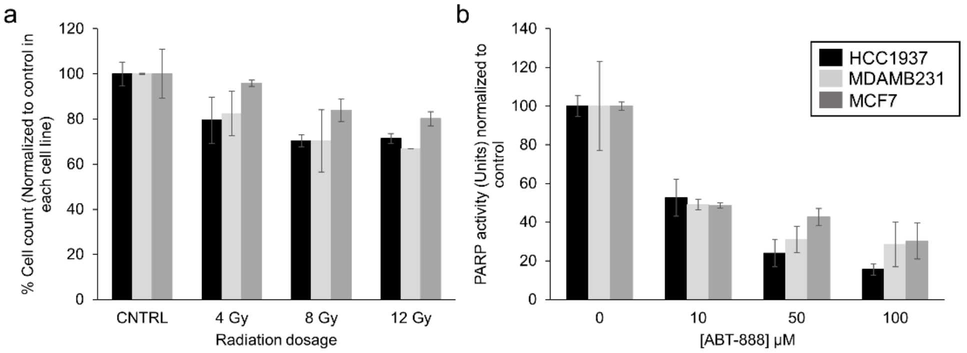

Supplementary Figure 1: Dose sensitivity and PARP activity analysis in breast cancer cells. a) Effect of dose of radiation on survival of HCC1937, MDAMB231 and MCF7 cells. Breast cancer cells were seeded in 6 well plates and cultured until they were ~80% confluent. Cells were irradiated with a ^{137}Cs source at a dose rate of 221 rad/min with different doses. Cell count was performed using a hemocytometer. b) Effect of different concentrations of PARPi ABT-888 on PARP's catalytic activity was studied using a chemiluminescent PARP activity assay. Breast cancer cells were cultured in 6 well plates until they were ~80% confluent after which the cells were treated with respective concentrations of ABT-888. The detailed procedure for measuring PARP activity is described in methods. Shown in the figure is the PARP activity in presence of activated DNA and different concentrations of ABT-888. The data are normalized to a control (DMSO) for each cell line. Data shown represent three biological replicates and the error bars indicate standard deviations.

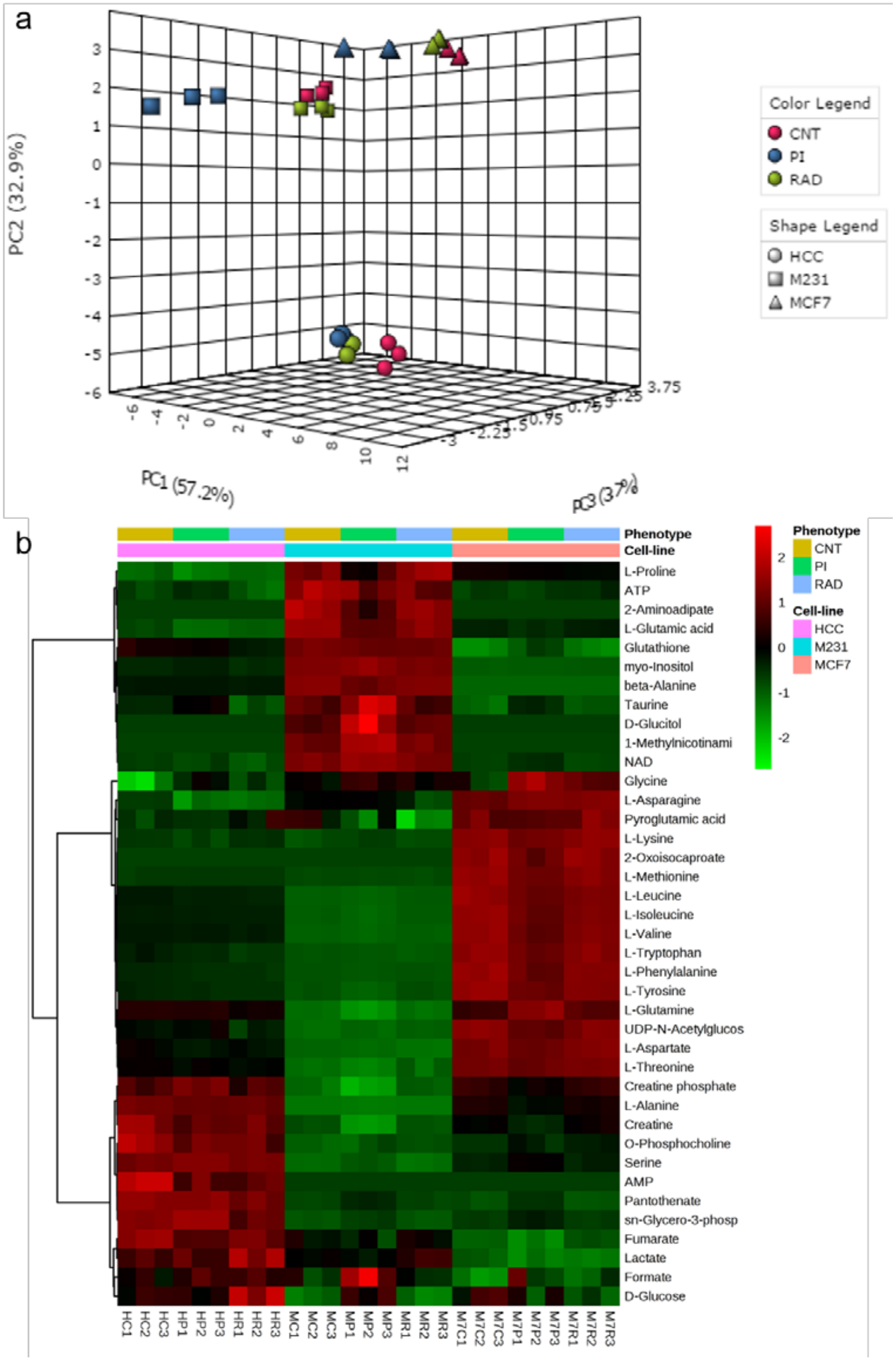

Supplementary Figure 2: Global analysis of metabolic profiles for the three breast cancer cell lines. a) 3D PCA plot showing three cell lines and treatment condition together using Two-factor analysis module in MetaboAnalyst. b) Heatmap of metabolite concentrations (auto-scaled) for all the cell lines and different phenotypes. Abbrev: CNT: control, PI: ABT-888, RAD: radiation, HCC: HCC1937, M231: MDAMB231.

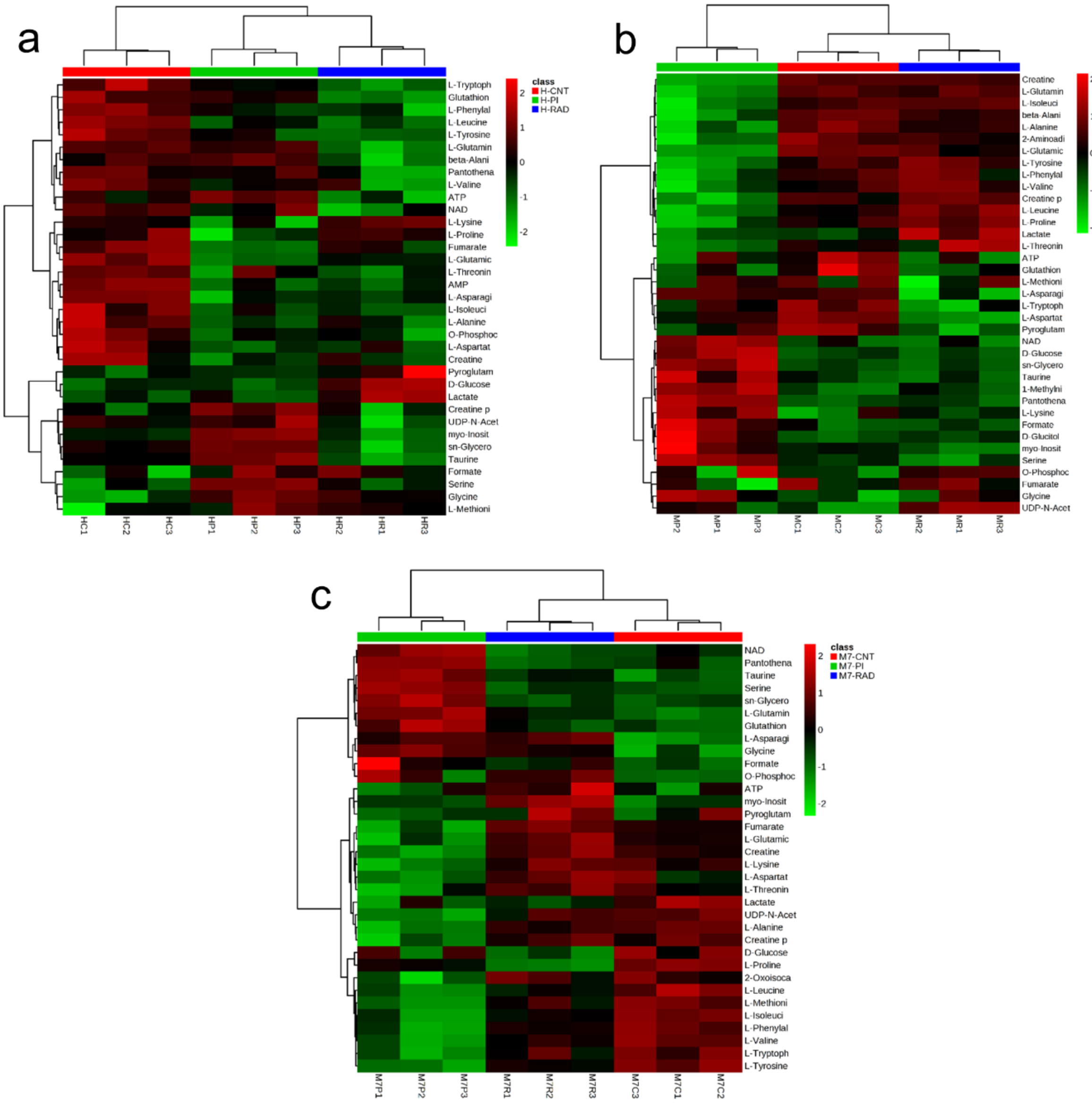

Supplementary Figure 3: Hierarchical clustering analysis for studying effect of radiation or PI in breast cancer cells. Hierarchical clustering was performed on the metabolite concentration data from a) HCC1937 cells, b) MDAMB231 cells, and c) MCF7 cells which treated with DMSO control, 50 µM ABT-888 or 8 Gy radiation. The concentration matrix was auto-scaled prior to clustering and the clustering was performed using Pearson's distance measure and ward linkage. Abbreviations: H: HCC1937, M: MDAMB231, M7: MCF7, CNT: control, PI: PARP inhibition, RAD: radiation.

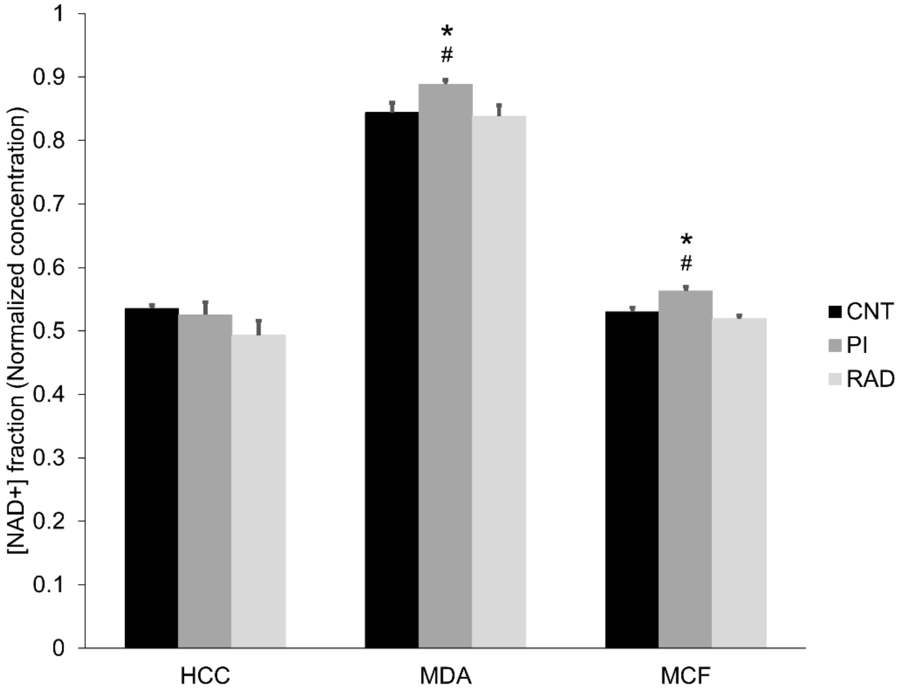

Supplementary Figure 4: Effect of PI on NAD concentration. The normalized NAD concentrations following PI or radiation treatment in HCC1937, MDAMB231 and MCF7 cell lines. Significance was measured by ANOVA with Tukey's HSD as post hoc. Abbreviations: HCC: HCC1937, MDA: MDAMB231, MCF: MCF7, CNT: control, PI: PARP inhibition, RAD: radiation. *FDR<0.05 relative to control and #FDR<0.05 relative to radiation.

Supplementary Table 1: Groups of metabolites identified and analyzed in this study

Group	Metabolites	Total
Essential amino acids	Isoleucine, Leucine, Lysine, Methionine, Phenylalanine, Threonine, Tryptophan, Valine, Tyrosine	9
Non-essential amino acids#	Alanine, Glutamate, Glutamine, Glycine, Asparagine, Aspartate, Proline, Serine	8
Central Carbon	Fumarate, Glucose, Lactate	3
Nucleotides	1-methylnicotinamide*, AMP***, ATP, NAD	4
Osmolytes	Myo-inositol, Sn-glycerol-3-phosphocholine, Taurine, Sorbitol (glucitol)*	4
Others	2-aminoadipate*, 2-oxoisocaproate**, Creatine, Creatine phosphate, Formate, Pantothenate, Pyroglutamate, UDP-N-Acetylglucosamine, Beta-alanine****	9
Phosphocholine	O-phosphocholine	1
Glutathione	Glutathione	1

Present in detectable amount exclusively in *MDAMB231 cells, **MCF-7 cells, ***HCC1937 cells and ****in both MDAMB231 and HCC1937 cell lines but not in the MCF7 cell line
